# Adjunctive Treatment with Subconjunctival and Topical Bevacizumab to Prevent Recurrence after Excision of Primary Pterygium: A Multi-centric Trial

**DOI:** 10.22336/rjo.2026.54

**Published:** 2026

**Authors:** Priyanka Pantola, Sandeep Gupta, Gagandeep Kaur, Pawan Kumar, Meenakshi Pathania, Priya Taank

**Affiliations:** 1 Command Hospital Air Force, Bangalore, India; 2 Command Hospital (CC), Lucknow, India; 3 Military Hospital, Dehradun, India; 4 Akash Institute of Medical Sciences and Research Centre, Devenhalli, Bangalore, India; 5 Military Hospital, Jammu, India; 6 Command Hospital (NC), Udhampur, India

**Keywords:** pterygium, recurrence of pterygium, conjunctival autograft, subconjunctival bevacizumab, topical bevacizumab, VEGF = Vascular endothelial growth factor, BVZ = Bevacizumab, CAG = Conjunctival autograft, 5-FU = 5-Fluorouracil, BCVA = Best corrected visual acuity, GAT = Goldmann Applanation tonometer, MMP-1 = Matrix metalloproteinase-1, LSCD = Limbal stem cell deficiency, HTFs = Human Tenon’s fibroblasts, LCAT = Limbal conjunctival autograft transplantation

## Abstract

**Objective:**

Pterygium is a common ocular condition, and excision with conjunctival autografting is the standard treatment. Recurrence after surgery is a known complication, and various adjunctive modalities are used along with excision to reduce recurrence. The use of vascular endothelial growth factor (VEGF) inhibitors, such as bevacizumab (Avastin), has been shown to reduce recurrence in several studies. This study was conducted to evaluate the efficacy of subconjunctival and topical Bevacizumab (BVZ) in preventing recurrence after pterygium surgery.

**Methods:**

This was a multi-center randomized controlled trial in which 1,800 patients were recruited and divided into three groups. In Group C, standard treatment of pterygium excision and conjunctival autografting was performed. Additionally, in Group A, subconjunctival BVZ was administered post-operatively and again 1 week after surgery, whereas in Group B, topical BVZ was administered for 4 weeks post-operatively. Recurrence was assessed at six and twelve months, along with visual acuity and complication rates.

**Results:**

Out of 1,800 patients recruited, 332 patients (18.44%) were lost to follow-up. Visual acuity in all three groups showed significant improvement at six and twelve months. The recurrence rate in Groups A and B was significantly lower than in Group C at both the six- and twelve-month follow-ups. The complication rates among the three groups were comparable.

**Discussion:**

The findings demonstrate that both subconjunctival and topical bevacizumab significantly reduce pterygium recurrence when used as adjuncts to conjunctival autografting, without increasing complications. These results support the role of VEGF inhibition in limiting postoperative fibrovascular proliferation and are consistent with previous studies. Although follow-up losses occurred, the large multicenter design strengthens the reliability and clinical applicability of the findings.

**Conclusions:**

When used as an adjunct, both subconjunctival and topical bevacizumab reduce the recurrence rate following primary pterygium excision with conjunctival autografting.

## Introduction

Pterygium is a fleshy growth arising from the conjunctiva that extends into the cornea. Common presenting complaints include redness, irritation, watering, blurring of vision, and cosmetic issues. Prevalence varies with geographic location and exposure to ultraviolet radiation. As per a meta-analysis, the global prevalence is approximately 12% [1]. Other risk factors include outdoor occupations, older age, and rural residence [2].

The standard treatment for primary pterygium is excision along with a conjunctival autograft (CAG). However, even with an autograft, recurrence can occur and is due to fibroblast activation and proliferation [3]. Various methods have been studied to reduce recurrence, including the use of amniotic membrane, radiotherapy, mitomycin C, and 5-Fluorouracil (5-FU) [4,5].

Local inflammation leading to fibrovascular proliferation is important both in the pathogenesis of pterygium and its recurrence. Angiogenic factors, such as vascular endothelial growth factor (VEGF), are upregulated in pterygium tissue and play a key role in its pathogenesis [6].

Bevacizumab (Avastin) is a humanized, monoclonal antibody against VEGF. It prevents VEGF interaction with its receptors on the surface of endothelial cells. It has been used successfully in various trials as an adjunct in pterygium surgery to prevent recurrence [7].

This study was conducted to evaluate the effectiveness of subconjunctival and topical bevacizumab (BVZ) as adjunctive techniques to prevent recurrence in patients undergoing pterygium surgery with the standard technique.

## Methods

This study is a multi-center randomized controlled trial conducted across seven centers over a period of three years. The study was approved by the Institutional Ethics Committee, and written informed consent was obtained from all participants. Patients aged over 20 years with primary pterygium of Tan Grade 2 (episcleral vessels partially visible under body of pterygium) or Grade 3 (episcleral vessels underlying body of pterygium were totally obscured) (**Fig. 1,2**) and with a best corrected visual acuity (BCVA) of 6/60 or better were included in the study. Patients with recurrent pterygium, autoimmune disorders, a history of past intraocular surgery, or systemic contraindications to bevacizumab were excluded.

**Fig. 1:**
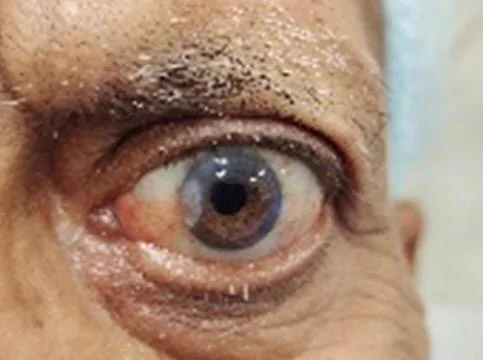
Grade 2 pterygium (Tan classification)

**Fig. 2:**
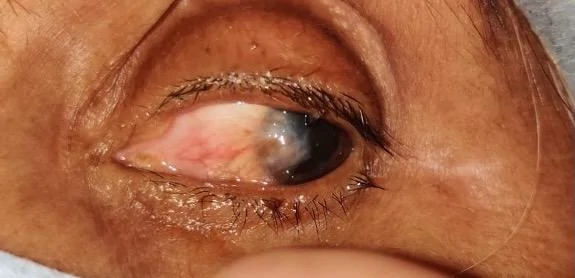
Grade 3 pterygium (Tan classification)

Considering the recurrence of pterygium in three independent treatment groups, the sample size was calculated using the formula for comparing two independent proportions, with a power of 0.80 and a significance level of 0.05. The mean calculated sample size was 454 participants per group. As this was a long-term, multicentric study, a 30% dropout rate was anticipated, and the final sample size was set at 600 patients per group.

A total of 1,800 patients were recruited, with 600 patients in each of the three groups. All patients underwent the following evaluations in the outpatient department:

1. Refraction and best-corrected visual acuity (BCVA) using the Snellen chart. Visual acuity was recorded and converted to the LogMAR scale.

2. Detailed slit-lamp examination and pterygium grading according to the Tan classification [8] (**Fig. 1,2**).

3. Anterior segment photography to document the grade and extent of the pterygium.

4. Dilated fundus evaluation was performed using a slit-lamp biomicroscope and indirect ophthalmoscopy.

5. Intraocular pressure measurement using a Goldmann Applanation tonometer (GAT).

Patients who met the inclusion criteria were randomly assigned to one of three groups using a computer-generated sequence of random numbers. All centers followed the same randomization protocol. The allocation sequence was concealed in an envelope, and the surgeon was informed of the patient’s group immediately before the surgical procedure.

Group A: Pterygium excision with conjunctival autograft and subconjunctival bevacizumab (BVZ) (Avastin, Genentech, South San Francisco, CA) 2.5 mg/0.1 ml postoperatively and repeated after 7 days (total cumulative dose - 5 mg).

Group B: Pterygium excision with conjunctival autograft and topical Bevacizumab (BVZ) 5mg/ml, 1 drop 4 times daily for 4 weeks postoperatively.

Group C: Pterygium excision with conjunctival autograft alone.

Patients were followed up on the 1st and 7th post-operative days, and subsequently at 1, 3, 6, and 12 months. On day 7 postoperatively, subconjunctival injection of bevacizumab was repeated in Group A, and compliance with topical bevacizumab drops was ensured in Group B. Patients in the topical BVZ group were instructed to collect freshly reconstituted drops at two weeks postoperatively.

During subsequent follow-up visits, refraction and best-corrected visual acuity (BCVA) were recorded, and any postoperative complications or recurrence were assessed. Recurrence was defined as any fibrovascular growth crossing the limbus and extending more than 1 mm onto the cornea. The primary outcome measure was recurrence at 6 months and 1 year. Visual outcome and complications were secondary outcome measures.

### Surgical technique - Pterygium Excision with Conjunctival Autograft using Fibrin Glue

All surgeries were performed under peribulbar anaesthesia (2% lignocaine + 0.5% Bupivacaine). The apex and body of the pterygium were dissected first. The dimensions of the scleral bed were measured, and a conjunctival autograft was harvested from the superotemporal conjunctiva. The graft was then secured onto the bare sclera with fibrin glue (Tisseel, Baxter) and allowed to dry for 1-2 minutes. At the end, preservative-free moxifloxacin drops were instilled, and a pressure patch was applied for 24 hours. Postoperatively, a combination eye drop containing moxifloxacin 0.5% and prednisolone acetate 1% was prescribed for one month – to be used every six hours for the first week, then tapered over the next three weeks. In addition, carboxymethylcellulose drops were administered every 6 hours for 4 weeks.

### Intervention in Group A

After securing the conjunctival graft with fibrin glue, patients in Group A were given a subconjunctival injection of bevacizumab (2.5 mg/0.1 ml) in the upper and lower bulbar conjunctiva, near the margins of the excised pterygium, using a 26 G needle. The same procedure was repeated 1 week postoperatively using topical proparacaine drops (total cumulative dose: 5 mg) (**Fig. 3A, B**).

**Fig. 3:**
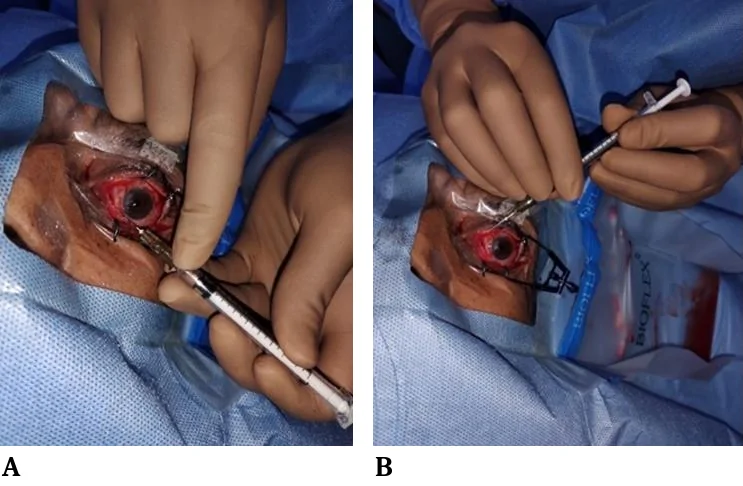
Subconjunctival Injection of Bevacizumab in upper and lower bulbar conjunctiva near margins of the excised pterygium

### Intervention in Group B

In patients in Group B, topical bevacizumab was prescribed from the first postoperative day (5 mg/ml) to be used four times a day for four weeks postoperatively. BVZ drops were freshly prepared from commercial Bevacizumab (25 mg/ml) by diluting it with normal saline (0.9%) under sterile conditions. Patients were instructed to store the drops in the refrigerator at 2-8^o^C.

### Statistical analysis

Data analysis was performed using SPSS software version 29.0. The one-way analysis of variance (ANOVA) and Chi-square test were used to compare the three groups for quantitative and qualitative data variables, respectively. A p-value < 0.05 was considered statistically significant.

## Results

A total of 1,800 patients were recruited (600 per group). Of these, 332 patients (18.44%) were lost to follow-up (84 patients in Group A, 141 patients in Group B, and 107 patients in Group C). However, this had been accounted for during the sample size calculation.

The three groups did not differ significantly in terms of gender, age, or grade of pterygium. The demographic characteristics of the study population are presented in **Table 1**. The mean age of patients in Groups A, B, and C was 45.24, 47.11, and 46.65 years, respectively. The overall mean age was 46.3 years, with a range of 21-81 years.

**Table 1:** Demographic characteristics of the study population

	Group A (n=516)	Group B (n=459)	Group C (n=493)
**Gender**			
**Male**	289 (56.01%)	271 (59.04%)	262 (53.14%)
**Female**	227 (43.99%)	188 (40.96%)	231 (46.86%)
**Age (years)**			
**< 30**	32 (6.2%)	16 (3.49%)	45 (9.13%)
**31-50**	323 (62.6%)	275 (59.91%)	268 (54.36%)
**51-70**	149 (28.88%)	166 (36.17%)	177 (35.9%)
**> 70**	12 (2.33%)	2 (0.44%)	3 (0.61%)
**Grade**			
**Grade 2**	139 (26.94%)	136 (29.63%)	140 (28.4%)
**Grade 3**	377 (73.06%)	323 (70.37%)	353 (71.6%)

The baseline visual acuity did not differ significantly among the three groups. Using ANOVA, there was a significant improvement in visual acuity across all three groups at 6 months and 1 year compared with baseline (P < 0.005). Using Tukey’s post hoc test for group-wise comparison, the improvement in visual acuity in Group A was found to be significantly greater than in Groups B and C at both 6 and 12 months (P < 0.001). **Fig. 4** illustrates the LogMAR visual acuity in the three groups at baseline, 6 months, and 1 year.

**Fig. 4:**
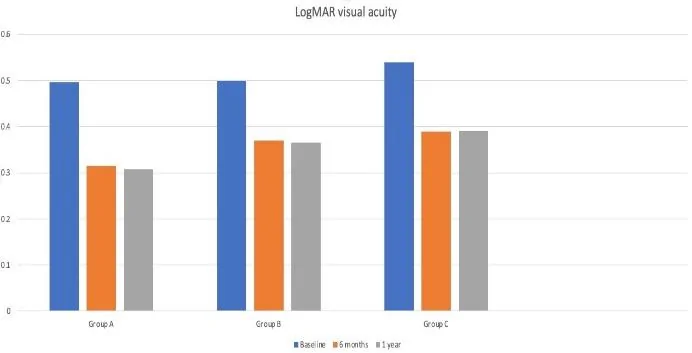
LogMAR visual acuity in the three groups

The recurrence rates in Groups A, B, and C were 8.33%, 9.15%, and 16.02% at 6 months and 8.91%, 9.59%, and 16.84% at 1 year, respectively (**Fig. 5**). At both 6 months and 1 year, the recurrence rates in Group A and B were lower than in Group C. Using the ANOVA test, this difference was found to be statistically significant (P < 0.001). Although the recurrence rate was lower in the subconjunctival BVZ group compared with the topical BVZ group, this difference was not statistically significant (P > 0.05).

**Fig. 5:**
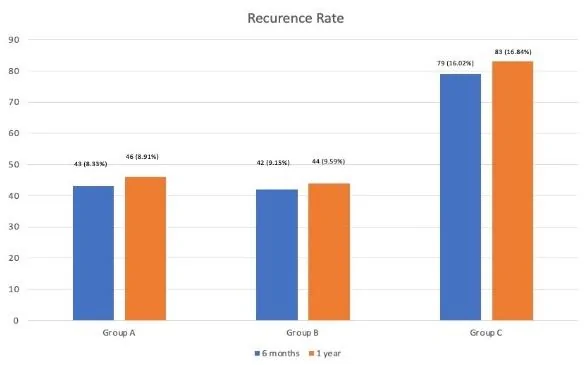
Recurrence rate at 6 months and 1 year postoperatively in the three groups

Complication rates in the three groups were 16.47%, 15.47%, and 14.60%, respectively. No graft dislocations were observed. There was no statistically significant difference among the three groups in terms of complication rates. Graft edema and conjunctival cysts were more frequently observed in Group A, whereas graft retraction was more common in Group B. The complication rates in the three groups are summarized in **Table 2**.

**Table 2:** Complication rates in the three groups

Complication	Group A (n=516)	Group B (n=459)	Group C (n=493)
**Subconjunctival hemorrhage**	35 (6.78%)	30 (6.54%)	30 (6.09%)
**Graft edema**	31(6.01%)	24 (5.23%)	27 (5.48%)
**Graft retraction**	15 (2.91%)	16 (3.49%)	12 (2.43%)
**Conjunctival cyst**	4 (0.78%)	1 (0.22%)	3 (0.61%)

## Discussion

Pterygium is common in Armed Forces personnel due to increased outdoor activities and prolonged exposure to sunlight in certain areas. The use of a conjunctival autograft is the standard treatment, which helps reduce recurrence. However, even with an autograft, the recurrence rate can be as high as 24.3% [9].

Various factors have been implicated in the pathogenesis of primary and recurrent pterygium, including increased expression of matrix metalloproteinase-1 (MMP-1) and vascular endothelial growth factor (VEGF). In addition, younger age and increased preoperative and postoperative ocular surface inflammation can also lead to recurrence [10]. Inflammation resulting from the surgical procedure itself can be a trigger.

Another important contributor is limbal stem cell deficiency (LSCD), as limbal stem cells form a physiological barrier that prevents conjunctivalization of the cornea. When these cells are damaged, fibrovascular proliferation can occur [11].

As previously discussed, Vascular Endothelial Growth Factor (VEGF) plays a key role. It also promotes fibrosis by stimulating angiogenesis. In a study by Park et al. [12], investigating the effect of BVZ on cultured human Tenon’s fibroblasts (HTFs), a dose-dependent inhibitory effect of BVZ on fibroblasts was demonstrated in both primary and recurrent pterygium. Moreover, HTF-produced matrix metalloproteinase-1 (MMP-1) levels decreased in a dose-dependent manner following BVZ exposure. Similar results were reported by O’Neill et al. [13], who observed that, in addition to its anti-angiogenic effect, BVZ also inhibits fibroblast proliferation and induces fibroblast cell death.

Bevacizumab (BVZ), administered either as a subconjunctival injection or topical drops, has been shown in various trials to prevent recurrence after pterygium surgery. The main objective of this study was to determine whether the adjunctive use of subconjunctival and topical BVZ reduces the recurrence rate.

In our study, the percentage of males was higher across all three groups, with Group A, B, and C comprising 56.01%, 59.04%, and 53.14% males, respectively. This finding was similar to that of Khanna et al. [14], who reported that male gender was associated with an increased risk of pterygium.

The overall mean age of patients included in the study was 46.3 years, with a range of 21 to 81 years. The highest prevalence was observed in the 31-50-year age group, followed by the 51-70-year age group. These results are comparable to those of a study by Maramula et al. [2], in which the mean age of participants was 47.5 years, with a range of 30-102 years.

In our study, there was a significant improvement in visual acuity in all three groups at 6 and 12 months compared with baseline. Our findings are similar to those of Singh et al. [15], in which visual acuity remained unchanged or improved postoperatively in both the subconjunctival bevacizumab and standard treatment groups. The improvement in visual acuity was probably due to a reduction in astigmatism.

Studies on recurrence following bevacizumab use have reported contradictory results. In a meta-analysis by Sun et al. [16], the subconjunctival BVZ group showed a statistically significant reduction in recurrence rate in cases of primary pterygium. Nuzzi et al. [7] evaluated the efficacy of subconjunctival bevacizumab at a dose of 2.5 mg/0.1 ml. They found that six months after surgery, recurrence was lower in the BVZ group (7.14%) than in the control group (24.39%). This is similar to the results of our study, in which the 6-month recurrence rate after subconjunctival BVZ was 8.33%.

Kasetsuwan et al. [17] randomized patients to receive either topical bevacizumab 0.05% or placebo four times daily for three months as an adjunct following pterygium surgery. Recurrence was lower in the topical BVZ group than in the placebo group. The corneal recurrence rate in this study was 8.33% at 3 months, which is similar to our results.

In a study by Razeghinejad et al. [18], pterygium excision with a rotational conjunctival flap followed by subconjunctival BVZ on the day of surgery and on day 4 was compared to placebo. In this study, BVZ had no significant effect on the recurrence rate. Similarly, Karalezli et al. [19] reported that topical bevacizumab had no additional effect on postoperative recurrence after pterygium excision and limbal conjunctival autograft transplantation (LCAT).

The most common complication encountered in all three groups was subconjunctival hemorrhage, followed by graft edema. Singh et al. [15] reported similar findings in a study comparing subconjunctival bevacizumab with placebo following pterygium surgery. Nuzzi et al. [7] also reported no complications related to bevacizumab use pre- or postoperatively after pterygium excision. A meta-analysis by Hu et al. [20], assessing the efficacy and safety of bevacizumab in the treatment of pterygium, found no statistically significant complications related to bevacizumab. However, the bevacizumab group was associated with an increased risk of subconjunctival hemorrhage. This is similar to what we have reported in our study. In our study, no local or systemic adverse events were observed with bevacizumab. The use of BVZ is also cost-effective in resource-limited settings, as a multi-dose vial can be used for multiple patients under strict aseptic precautions.

This study is unique in that it compares three different treatment modalities in a diverse group. However, it was limited by a high number of patients lost to follow-up due to the COVID-19 pandemic. The trial’s limitation is the inability to achieve assessor blinding because some participating centers were single-specialty.

## Conclusion

In conclusion, both subconjunctival injection and topical application of bevacizumab are effective adjuncts for reducing pterygium recurrence and may be used routinely without significant local or systemic complications.
